# Faculty knowledge and attitudes regarding predatory open access journals: a needs assessment study

**DOI:** 10.5195/jmla.2020.849

**Published:** 2020-04-01

**Authors:** Stephanie M. Swanberg, Joanna Thielen, Nancy Bulgarelli

**Affiliations:** Associate Professor and Information Literacy & eLearning Librarian, Medical Library, Oakland University William Beaumont School of Medicine, Rochester, MI, swanberg@oakland.edu, https://orcid.org/0000-0003-0553-8339; Biomedical Engineering Librarian, Art, Architecture & Engineering Library, University of Michigan, Ann Arbor, MI, jethiele@umich.edu, http://orcid.org/0000-0002-2983-5402; Associate Professor and Director, Medical Library, Oakland University William Beaumont School of Medicine, Rochester, MI, bulgarel@oakland.edu

## Abstract

**Objective:**

The purpose of predatory open access (OA) journals is primarily to make a profit rather than to disseminate quality, peer-reviewed research. Publishing in these journals could negatively impact faculty reputation, promotion, and tenure, yet many still choose to do so. Therefore, the authors investigated faculty knowledge and attitudes regarding predatory OA journals.

**Methods:**

A twenty-item questionnaire containing both quantitative and qualitative items was developed and piloted. All university and medical school faculty were invited to participate. The survey included knowledge questions that assessed respondents’ ability to identify predatory OA journals and attitudinal questions about such journals. Chi-square tests were used to detect differences between university and medical faculty.

**Results:**

A total of 183 faculty completed the survey: 63% were university and 37% were medical faculty. Nearly one-quarter (23%) had not previously heard of the term “predatory OA journal.” Most (87%) reported feeling very confident or confident in their ability to assess journal quality, but only 60% correctly identified a journal as predatory, when given a journal in their field to assess. Chi-square tests revealed that university faculty were more likely to correctly identify a predatory OA journal (*p*=0.0006) and have higher self-reported confidence in assessing journal quality, compared with medical faculty (*p*=0.0391).

**Conclusions:**

Survey results show that faculty recognize predatory OA journals as a problem. These attitudes plus the knowledge gaps identified in this study will be used to develop targeted educational interventions for faculty in all disciplines at our university.

## INTRODUCTION

Open access (OA) publishing allows free, unlimited online access to scholarly literature without the paywalls associated with traditional publishing models [1]. Legitimate OA journals uphold rigorous peer review, allow authors to retain copyright, and have been shown to increase an author’s visibility [2, 3]. However, an unanticipated effect of this model has been the emergence of predatory OA journals that “exist for the sole purpose of profit, not the dissemination of high-quality research findings and furtherance of knowledge” [4]. These journals attempt to entice authors into paying an article processing fee and never publish the article or publish the article rapidly without quality peer review. This practice significantly impacts the quality of research being published and could have detrimental implications for authors, their institutions, and their fields [5–8].

To date, most of the literature on predatory OA journals has been opinion-based (editorials, commentaries, news items, and so on), aimed at raising awareness of and cautioning against publishing in predatory OA journals [5, 9–14]. Numerous investigations of faculty attitudes regarding OA in general have been published [15–20], many revealing a general skepticism toward legitimate OA journals [16, 17, 20–22]. Only a handful of investigations have focused on predatory OA journals specifically, often assessing faculty attitudes [7, 8, 23–25]; fewer still have tested faculty knowledge of predatory OA journals directly [7, 23]. Libraries currently do and will continue to play a pivotal role in educating users about predatory OA journals [4, 26]. Therefore, it is important to understand faculty’s baseline knowledge of and attitudes toward predatory OA journals.

Since 2016, the Oakland University William Beaumont School of Medicine Library (OUWB Medical Library) has spearheaded initiatives to raise awareness of predatory OA journals among faculty and students. Specifically, these journals have been discussed in faculty development sessions about publishing and journal selection, in the medical school curriculum as part of the required research program, and in three dedicated sessions on predatory OA journals during Oakland University Libraries’ (OU Libraries’) annual OA Week events. In addition, the OUWB Medical Library tracks institutional scholarship and has noted several instances of faculty publishing in questionable or known predatory OA journals.

Recently, OU Libraries included assessing faculty knowledge of predatory OA journals as a top priority in their strategic plan. As a result, librarians from the university and medical school libraries collaborated to conduct an educational needs assessment to investigate and compare current knowledge of and attitudes toward predatory OA journals among university and medical school faculty. Our research questions were:

1. What gaps, if any, exist in faculty members’ knowledge of predatory OA journals, including the ability to identify one?
2. What are faculty attitudes regarding predatory OA journals?

The results of this project will be instrumental in tailoring faculty development sessions at the university that address identified knowledge gaps and attitudes and may provide a framework for other libraries to perform similar assessments at their own institutions.

## METHODS

As no previous surveys have been published to date on predatory OA journals specifically, the authors developed a twenty-item Qualtrics (Qualtrics XM, Provo, UT, USA) questionnaire (supplemental Appendix A). The survey included both quantitative and qualitative items and was divided into several sections:

- demographic items including status as a university or medical school faculty member, rank, department, field of study, and total number of career publications
- previous training on predatory OA journals
- knowledge of predatory OA journals including assessing respondents’ ability to: (1) distinguish between characteristics of predatory and legitimate OA journals and (2) identify a predatory OA journal when asked to review the website of one in their field; we selected four predatory OA journals in various fields that had a polished appearance to encourage a thorough evaluation
- attitudes about predatory OA journals, including confidence in identifying predatory OA journals and the importance of discussing such journals at institutional and professional levels
- previous experiences with predatory OA journals
- resources used to assess journal quality
- how libraries can assist with assessing journal quality

Prior to institutional review board (IRB) review, the survey was pilot-tested by 9 faculty members and revised. This study was deemed exempt by the Oakland University IRB. Respondents needed to be current (full- or part-time) or emeritus faculty members at Oakland University or OUWB School of Medicine to be eligible for the study. The screening questions to determine eligibility were required, but respondents could elect to skip any subsequent questions.

All eligible university (n=1,669) and medical school (n*=*1,425) faculty were invited to participate in the study via emails through university email discussion lists in early February 2019. The emails were sent to university faculty via the Oakland University Provost’s Office and to medical faculty via the OUWB Office of Faculty Affairs. A reminder email was sent in late March 2019, and the survey was closed at the end of April 2019. Descriptive statistics were used to summarize the data, and comparisons between groups were analyzed using chi-square tests. A *p*-value <0.05 was considered statistically significant. All data analysis was completed using SAS 9.4 (SAS Institute, Cary, NC, USA).

## RESULTS

A total of 220 responses were collected. Of these, 34 were removed because respondents did not proceed beyond the screening questions, and 3 were removed because the respondents were not faculty members. Therefore, a total of 183 faculty members completed the survey, representing an overall response rate of 5.9%.

### Demographics

As all survey questions were optional, 17 respondents elected not to respond to the demographic questions. Of the respondents, 37.4% (n=62/166) were medical school faculty and 62.7% (n=104/166) were university faculty. The majority of respondents held rank as associate professors (36.3%), followed by assistant professors (26.9%), and professors (16.9%). For university faculty, the largest number of respondents came from social and behavioral science disciplines (32.0%), followed by the sciences (15.5%), humanities (14.6%), and business (9.7%), with all university schools and colleges represented. For medical school faculty, basic science faculty accounted for the most respondents (24.6%), followed by internal medicine (13.1%), with small numbers from various clinical departments including emergency medicine, family medicine, orthopedic surgery, pediatrics, radiation oncology, and surgery. Supplemental Appendix B provides a complete breakdown of respondents by rank and department.

**Figure 1:**
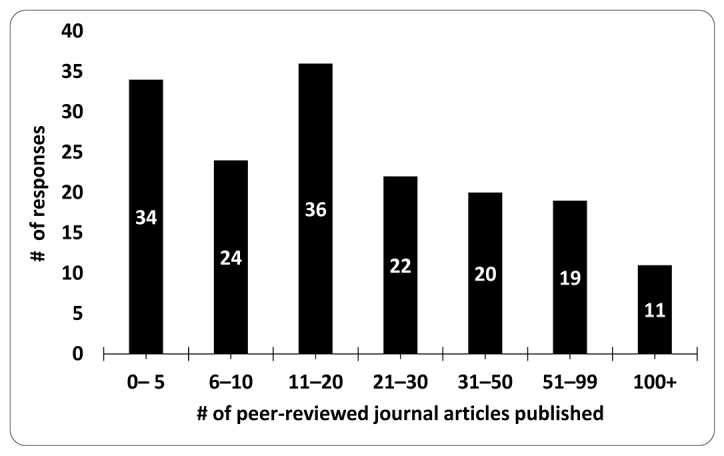
Respondents’ number of peer-reviewed journal articles published in their careers

Most respondents had published between 11 and 20 peer-reviewed publications in their academic careers (21.7%; n=36), closely followed by 0–5 (20.5%; n=34) publications (Figure 1).

### Previous training on predatory open access (OA) journals

Only a few respondents (13.3%, n=22) reported receiving training on predatory OA journals. Table 1 shows the type of training reported by respondents.

**Table 1:** Respondents’ training on predatory open access (OA) journals (multiple choices allowed)

Type of previous training	n
Library workshop at Oakland University (OU) or Oakland University William Beaumont School of Medicine Library (OUWB)	14
Library workshop at another institution	7
Professional conference	6
Workshop in their department	4
Previous coursework	3
Webinar	2

### Knowledge of predatory OA journals

To answer the first research question, all 183 respondents were asked questions to assess their knowledge (or lack thereof) of predatory OA journals. When respondents were asked if they had previously heard of the term “predatory OA journal,” most (70.5%, n=129) reported “yes,” but a substantial portion reported “no” (23.0%, n=42) or “unsure” (6.6%, n=12). One respondent commented, “Wow, I had never heard of predatory [OA] journals so I am glad that this survey called the issue to my attention.”

**Figure 2:**
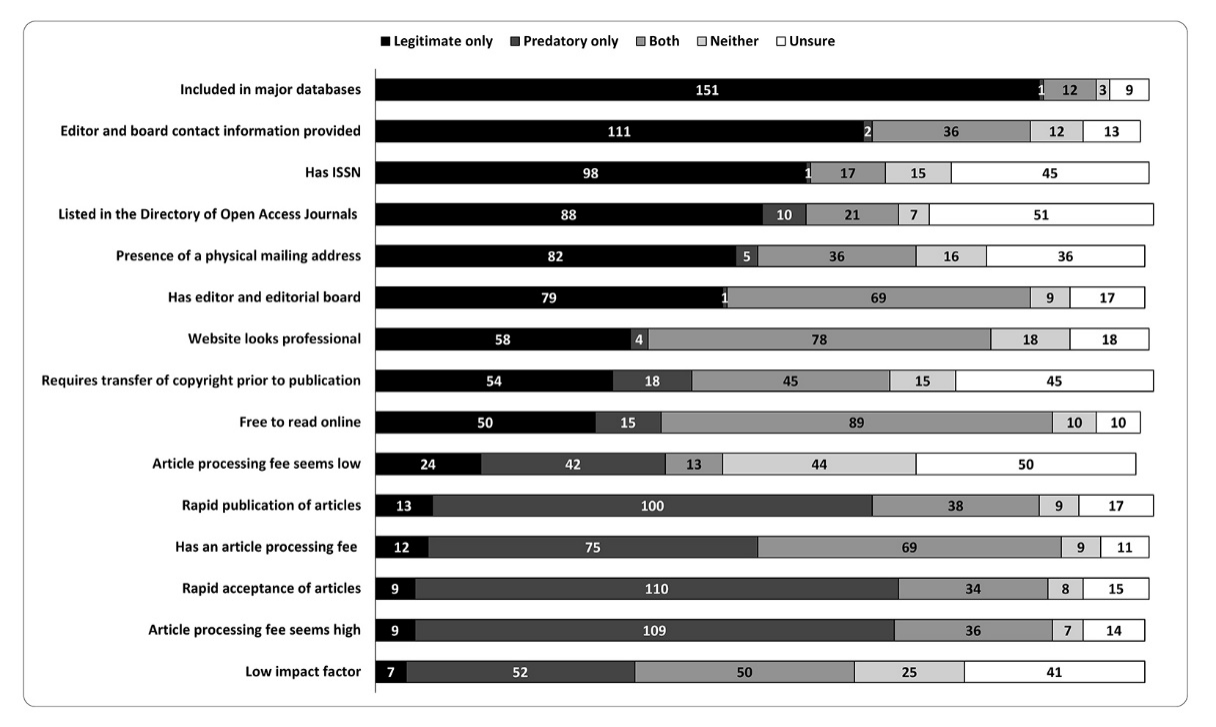
Respondents’ identification of journal characteristics as associated with legitimate or predatory open access (OA) journals

When participants were asked to review a series of OA journal characteristics and identify which ones they associated with legitimate OA journals, predatory OA journals, both, neither, or unsure, several trends emerged (Figure 2). Most respondents correctly identified that legitimate OA journals can be indexed in major databases (85.8%; n=151/176), but only about half identified a journal being listed in the Directory of Open Access Journals (DOAJ) (49.7%; n=88/177) as a characteristic of this type of journal. As for characteristics of predatory OA journals, some respondents correctly identified 2 characteristics: rapid acceptance of articles (62.5%; n=110/176) and rapid publication of articles (56.5%; n=100/177). Only half of respondents correctly identified being free to read online (51.1%; n=89/174) as a characteristic of both legitimate and predatory OA journals. There were a number of characteristics that faculty were unsure about:

- journal being listed in the DOAJ (28.8%; n=51/173)
- article processing fee seems low (28.9%; n=50/173)
- requirement of transfer of copyright prior to publication (25.4%; n=45/176)
- an International Standard Serial Number (ISSN) (25.6%; n=45/176)

Many of these characteristics could be associated with either legitimate or predatory OA journals. Therefore, responding “both” for these questions indicated an understanding that these characteristics were not absolute indicators of legitimacy.

When comparing university faculty to medical faculty responses to these journal characteristics, some statistically significant differences were found. For “rapid acceptance of articles,” a larger proportion of medical faculty (35.5%) than university faculty (18.3%) stated that this was a characteristic of a legitimate OA journal (χ^2^(1)=6.19, *p*=0.0129). There was no significant difference in responses to “rapid publication of articles” between groups (*p*>0.05). For “presence of physical mailing address” on the journal website, a larger proportion of medical faculty (82.3%) than university faculty (60.6%) stated that this was a characteristic of a legitimate OA journal (χ^2^(1)=8.49, *p*=0.0036). However, a larger proportion of medical faculty (33.9%) than university faculty (15.4%) stated this same characteristic was associated with a predatory OA journal (χ^2^(1)=7.66, *p*=0.0056). For “journal has low impact factor,” a larger proportion of medical faculty (74.2%) than university faculty (48.1%) stated that this was a characteristic of a predatory OA journal (χ^2^(1)=10.86, *p*=0.0010). Furthermore, for “listed in the Directory of Open Access Journals,” a larger proportion of university faculty (6.7%) than medical faculty (0) believed that being listed in the DOAJ was a characteristic of neither type of OA journal (χ^2^(*1*)=*4.36, p*=0.0369).

### Assessment of the quality of an OA journal

Respondents were asked which of 5 broad disciplinary fields (arts, humanities, medicine and health sciences, sciences, or social and behavioral sciences) they felt most comfortable with in assessing the quality of a journal. They were then asked to assess the website of an actual predatory OA journal in their chosen fields and to give their opinion on whether it was a predatory or legitimate OA journal. In total, only 60.0% (n=103/172) correctly identified the journal as predatory, with 15.1% (n=26/172) incorrectly determining it was legitimate, and 25.0% (n=43/172) indicating they were unsure. A significantly larger proportion of university faculty (69.2%) than medical faculty (43.6%) correctly identified the journal as predatory (χ^2^(1)=14.88, *p*=0.0006). Faculty who self-identified (in the demographics section) as being in the social and behavioral sciences discipline were the only discipline significantly more likely to correctly identify the journal as predatory (χ^2^(2)=6.24, *p*=0.0442).

There were no significant differences (*p*>0.05) in faculty members’ ability to identify the predatory OA journal based on their rank, previous training, or publication history. There were also no significant differences (*p*>0.05) among the 4 predatory OA journals used in the survey in terms of faculty correctly identifying the journal as predatory. Faculty who selected to assess the journal used for both the humanities and social sciences categories had the greatest success at identifying it as predatory, 75.0% (n=15/20) and 70.6% (n=36/51), respectively; the sciences journal at 60.7% (n=17/28) and medicine and health sciences journal at 48.6% (n=34/170) followed. Only 2 respondents selected to assess the arts journal, with 50.0% success (n=1/2) in correctly identifying it as predatory.

When respondents were asked to give at least two reasons why they believed the journal was predatory or legitimate, common themes emerged. Reasons for identifying it as predatory included promise of rapid review and/or publication, journal scope too broad, information about the editorial board missing, and only Bitcoin accepted as payment. Reasons for identifying it as legitimate included presence of an ISSN, peer review process described, digital object identifiers (DOIs) for articles, impact factor listed on website, editorial board named, and legitimate appearance of website.

### Previous experience with predatory OA journals

The majority of respondents had some previous experience with predatory OA journals (Table 2). When comparing university and medical faculty, the only significant finding was that medical faculty were more likely to have been asked to serve on the editorial board of a predatory OA journal (61.3%) than university faculty (42.3%; χ^2^(1)=6.00, *p*=0.0180).

**Table 2:** Respondents’ previous experience with predatory OA journals (multiple choices allowed)

Experience with predatory OA journals	n (total=183)	%
Asked to publish in a predatory OA journal	133	72.6%
Asked to serve on an editorial board	83	45.4%
Receive unsolicited email solicitations a few times per month	71	38.8%
Asked to serve as a peer reviewer	56	30.6%
Receive unsolicited email solicitations a few times per week	37	20.2%

When asked if they had previously published in a predatory OA journal, 7 of the 169 respondents who answered this question said “yes,” and 11 were “unsure.” The top reasons given for publishing in a predatory OA journal (multiple choices allowed) were: not aware of it being a predatory OA journal (n=10), could not get article published elsewhere (n=6), and option for rapid acceptance and/or publication (n=6).

### Attitudes about predatory OA journals

**Figure 3:**
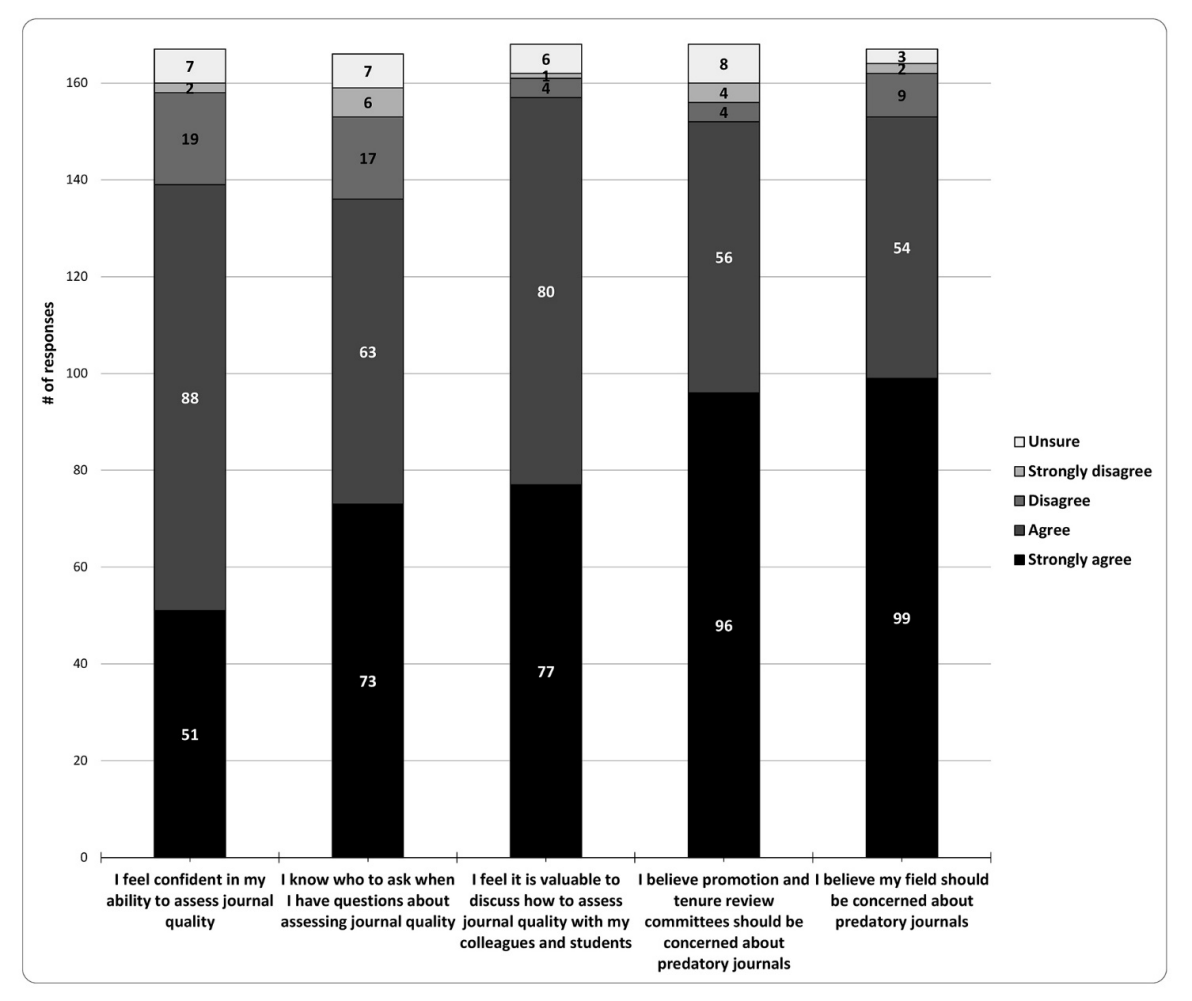
Respondents’ agreement with statements about their attitudes toward predatory OA journals

To answer the second research question, respondents were asked a series of Likert scale questions (Figure 3). When presented with the statement, “I feel confident in my ability to assess journal quality,” 86.9% (n=139/160) of faculty strongly agreed or agreed. When given the statement, “I believe promotion and tenure review committees should be concerned about predatory OA journals,” 95.0% (n=152/160) strongly agreed or agreed. In addition, 93.3% (n=153/164) strongly agreed or agreed with the statement “My field/profession should be concerned about predatory OA journals.” In the final open-ended question, several respondents shared concerns and thoughts about journal quality considerations in the promotion and tenure process:

“I think tenure and promotion committees should have to assess (or have the candidate assess) journal status.”

“This is an important issue for academic integrity, merit pay, and promotion & tenure decisions.”

“Given the increasing importance of number of publications published for tenure, it becomes more likely that desperate junior faculty will succumb to predatory [OA] journals. Frankly, that is becoming more of an issue across all faculty levels.”

In addition, several comments emerged about the importance of discussing predatory OA journals with students:

“I think it is important to teach students, as well as faculty, about these journals. Although some students’ awareness of varying quality in publications is excellent, many still assume that if it looks like a reputable, scholarly source, it is.”

“Having a module to train graduate students on how to identify predatory [OA] journals would be fantastic!”

“Training in this area should be part of the curriculum for all medical students, residents, and fellows.”

“I appreciate knowing that such journals exist and will look closely when advising my students.”

After being asked these Likert scale questions, respondents were asked to describe, in an open-ended format, why they did or did not feel confident in their ability to judge journal quality. Of those who were confident, their answers coalesced around several themes: knowing the reputable journals in their field, knowing which resources to use to assess journal quality, researching a journal prior to submission, and having previous training or consultations with librarians. Conversely, respondents who were not confident in their ability to assess journal quality cited several recurring themes: sophistication of many predatory OA journals, lack of experience or education, mistrust in the OA model in general, and the sheer number of OA journals. In fact, three respondents specifically commented in the final open-ended question about their fears of this phenomenon:

“This is a bit of a scary issue because many predatory [OA] journals seem legitimate.”

“I think this is a big issue that frankly scares me and has the potential to undermine our legitimacy as researchers.”

“Predatory [OA] journals are scary things.”

There were no significant differences in self-reported confidence in assessing journal quality based on faculty rank, previous training, publication history, or discipline. However, a larger proportion of university faculty (90.7%) than medical faculty (79.3%) reported higher self-confidence in their ability to assess journal quality (χ^2^(3)=8.36, *p*=0.0391). Of the respondents who strongly agreed or agreed (n=139) with the statement, “I feel confident in my ability to assess journal quality,” 66.0% were able to correctly identify a predatory OA journal in their fields, compared to 60.0% of all respondents. Of the respondents who strongly disagreed or agreed with the statement (n=21), 38.1% were able to correctly identify a predatory OA journal in their fields.

### Resources used to identify journal quality

Respondents were asked to select what resources they used to help assess the quality of journals when looking to publish their work. Of the 165 responses, the 3 highest reported resources were colleagues (76.4%; n=126); Google or another search engine (54.5%; n=90); and a professional email discussion list, blog, or website (42.4%; n=70). Some also used librarians (38.8%; n=64) or consulted the library’s website (19.4%; n=32).

### How libraries can assist faculty in assessing journal quality

**Figure 4:**
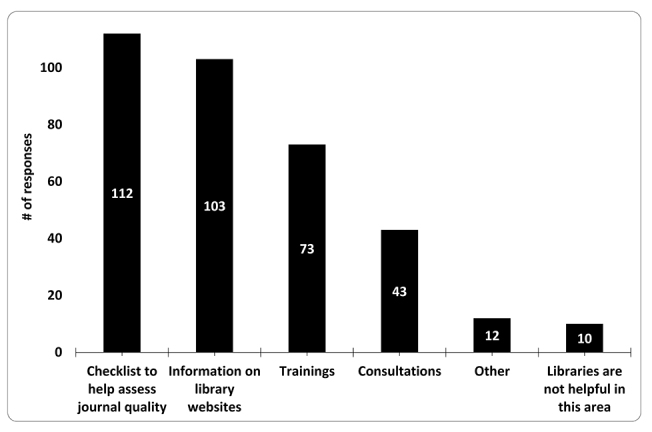
Respondents’ preferences for the Oakland University Libraries to assist in assessing journal quality (multiple choices allowed)

When asked what OU Libraries could do to assist them, faculty were interested in a checklist (70.9%; n=112/158); information on the libraries’ website (65.2%; n=103/158); workshops or training (46.2%; n=73/158); or individual consultations (27.4%; n=43/157) (Figure 4). A larger proportion of university faculty (85.3%) than medical faculty (62.9%) stated that their colleagues were a resource for assessing journal quality (χ^2^(1)=10.86, *p*=0.0010). In addition, several respondents in open-ended comments noted the aid of librarians: “I have found the OU librarians invaluable in assessing the legitimacy of online journals” and “The Library staff has been very helpful to me!” Ten respondents did not think the OU Libraries could be helpful in this area.

## DISCUSSION

Predatory OA journals are a pervasive issue across all of academia. However, to date, most of the published literature on predatory OA journals has been editorial in nature rather than methodical investigations [5, 9–14]. Previous studies have focused on faculty attitudes regarding OA journals in general [15–18], with only a handful studying predatory OA journals specifically [7, 19, 23–25]. This study assessed cross-disciplinary university and medical faculty members’ ability to identify predatory OA journals and sought to understand their attitudes and previous experiences with these journals.

Previous investigations into a particular group’s familiarity with predatory OA journals took different methodological approaches: talking to authors who had previously published in a predatory OA journal [23, 24], assessing the influence of predatory OA journals on clinical care [7, 8], or evaluating an educational intervention for early career researchers [25]. A study by Christopher and Young in 2015 that assessed veterinary and medical authors’ (students, residents, and faculty) knowledge of predatory OA journals before attending a scientific writing workshop found that only 23.0% of respondents were aware of the term “predatory OA journal,” and when asked in an open-ended response to define “predatory OA journal,” most participants associated it with poor quality rather than truly predatory practices [19]. The current study builds on Christopher and Young’s results by directly assessing faculty’s identification of the particular characteristics of legitimate versus predatory OA journals. Although a larger proportion of faculty (70.5%) in this study had heard of the term, many thought that some characteristics of the OA publishing model were, in fact, indicators of predatory OA journals, such as charging article processing fees.

We had anticipated that faculty would exhibit some knowledge gaps regarding predatory OA journals. However, we were surprised by the extent of these knowledge gaps, as only 60.0% of faculty were able to correctly identify a journal as predatory. Therefore, there was a mismatch between ability and self-reported confidence, as 86.9% of faculty reported that they strongly agreed or agreed with the statement, “I feel confident in my ability to assess journal quality.” Thus, these findings suggested that many faculty members continued to demonstrate gaps in knowledge about the OA publishing model, a phenomenon that has been widely reported in the literature [16, 17, 20–22]. Faculty respondents in this survey also indicated an overall lack of trust in the OA publishing model. One of the comments was particularly striking and illustrates ongoing confusion about the OA model:

“If the work cannot be published in a legitimate journal, it shouldn’t be published. I don’t understand faculty who do not understand that paying to publish is not consistent with the values and traditions of the academy.”

Two other recent studies investigated reasons why authors decided to publish in predatory OA journals: one through a survey [23] and the other through in-depth interviews [24]. Both found that authors self-reported similar reasons, including lack of awareness that the journal was predatory, pressure to publish, lack of confidence in their ability to publish in a high-quality journal, and lack of research experience [23, 24]. Though only a small portion of respondents admitted to publishing in a predatory OA journal in the current study, our results are consistent with these previous findings.

Based on earlier educational efforts at our university, we hypothesized that faculty in the sciences, including medicine and health sciences, would be more likely to correctly identify a sample predatory OA journal. However, we found that faculty in social and behavioral science disciplines were significantly more likely to correctly identify a predatory OA journal, with no other significant differences related to discipline, demonstrating that education and awareness of predatory OA journals needs to reach faculty in all disciplines.

Our respondents appeared to agree with this need, as 91.6% (n=153) strongly agreed or agreed with the statement, “I believe my field should be concerned about predatory OA journals.” In addition, there were no significant associations between faculty members’ ability to correctly identify a journal as predatory and their rank, publication history, or previous training. This finding supported those of a survey on OA publishing conducted at our institution in 2014, which concluded that predictions about faculty opinions on, use of, and publishing in OA journals could not be made based on age, rank, or seniority [15]. However, we found that university faculty were significantly more likely to report being more confident than medical faculty, which was interesting considering that most of the libraries’ educational activities about OA journals to date have been offered directly to medical school faculty at OUWB. This, again, points to the need to target educational efforts at faculty at all levels and disciplines and provides additional motivation for librarians to advocate for their role in education about predatory OA journals.

One of the major takeaways from the survey was the need for our 2 libraries to publicize and advocate for our educational role in this area and become recognized as a reputable resource to answer questions related to predatory OA journals. Unsurprisingly, most faculty reported colleagues (76.4%) and Google or another search engine (54.5%) as their top 2 sources when investigating journals in which to publish. Only 38.8% reported asking a librarian, and only 19.4% had consulted the library’s website.

However, it was clear that faculty were seeking help and more information about predatory OA journals, with most reporting wanting a checklist to assess journal quality (70.9%), followed by information on the library website (65.2%). Many also wanted the option of individual consultations and educational workshops for themselves and their students. Based on these results, we plan to create web pages dedicated solely to predatory OA journals on both the university and medical library websites, develop a series of trainings and workshops targeting all departments on campus, and more widely disseminate our locally developed journal authenticity checklist that is currently available on the OU Libraries’ website. In addition, linking to other freely available journal quality checklists, such as Think. Check. Submit and a recently published checklist by Blas et al. [27], would be beneficial for enhancing faculty skills in assessing journals [5].

In designing the survey, we made a conscious decision to not include predatory journal or publisher lists, because Beall’s List is no longer active and Cabell’s is not in the libraries’ budget. Furthermore, our libraries’ educational efforts have focused on developing critical thinking skills in appraising journal quality and legitimacy, which aligns with the Association for College & Research Libraries’ Framework for Information Literacy for Higher Education [28]. Other institutions could elect to add one or more of these lists to the survey, depending on their current subscriptions. As an initial step, we created an executive summary describing the project, major results, and future plans that was disseminated with permission from the provost’s office, medical school, and library administration to various stakeholders on campus, including library faculty liaisons, all medical school department chairs, the university research office, graduate office, and university research committee.

### Limitations

This study does have some limitations. First, the overall response rate of 5.9% was low, which was not unexpected. In the future, if university policy allows, incentives could be considered to increase the response rate. As faculty self-selected to participate in the survey, some selection bias might also be present, favoring those with a preexisting interest in or knowledge of the topic. However, most schools, colleges, and clinical departments were represented, and we had sufficient responses to conduct statistical analysis. Although the data might be representative and generalizable on a greater scale, a larger sample size and response rate could reveal additional differences. Second, we decided that the scope of this project would be on faculty knowledge and attitudes about predatory OA journals, so students and medical residents were excluded. Future studies could repeat the survey with a student or resident population to provide additional insights.

Publishing, knowingly or unknowingly, in a predatory OA journal can have serious implications for authors: it can affect their reputations as scholars, impact their ability to submit to other journals, and diminish the quality of their work. Some institutions assess the quality of journals that faculty choose to publish in and may use this as one criterion for hiring, promotion, and retention. Understanding faculty’s current knowledge levels is essential in developing targeted educational campaigns and professional development. By directly assessing faculty knowledge of such journals and their ability to identify them, in addition to their attitudes, this study provides a clearer understanding of the topic in this particular population. Overall, predictions could not be made about faculty knowledge or attitudes based on rank, discipline, publication history, or previous training about predatory OA journals. However, most faculty were interested in librarians’ help in selecting legitimate OA journals to publish in, providing an impetus for OU Libraries to promote their role in this process and tailor their educational initiatives to these needs.

## DATA AVAILABILITY STATEMENT

Data associated with this article are available in the OUR@Oakland Institutional Repository at http://hdl.handle.net/10323/6856.

## SUPPLEMENTAL FILES

Appendix A Full survey instrument

Appendix B Supplemental tables

## 

**Stephanie M. Swanberg, MSI, AHIP**, swanberg@oakland.edu, https://orcid.org/0000-0003-0553-8339, Associate Professor and Information Literacy & eLearning Librarian, Medical Library, Oakland University William Beaumont School of Medicine, Rochester, MI

Joanna Thielen, MSI, MS*, jethiele@umich.edu, http://orcid.org/0000-0002-2983-5402, Biomedical Engineering Librarian, Art, Architecture & Engineering Library, University of Michigan, Ann Arbor, MI

**Nancy Bulgarelli, MSLS**, bulgarel@oakland.edu, Associate Professor and Director, Medical Library, Oakland University William Beaumont School of Medicine, Rochester, MI
