# Supplementary material for: Faculty knowledge and attitudes regarding predatory open access journals: a needs assessment study
Source: J Med Libr Assoc. 2020 Apr 1;108(2):208–18. doi: 10.5195/jmla.2020.849 (PMC7069810; doi:10.5195/jmla.2020.849)
Supplement: Appendix A [file jmla-108-208-s001.pdf]

JMLA Journal of the Medical Library Association

SC
Supplemental content to
J Med Libr Assoc. Apr;108(2):dx.doi.org/10.5195/jmla.2020.849
www.jmla.mlanet.org
© Swanberg, Thielen, Bulgarelli 2020

Faculty knowledge and attitudes regarding predatory open access journals: a needs assessment study

Stephanie M. Swanberg, MSI, AHIP; Joanna Thielen, MSI, MS; Nancy Bulgarelli, MSLS

APPENDIX A

Full survey instrument

1. Have you previously heard of the term "predatory journal"?

- Yes
- No
- Unsure

According to a recent article, predatory journals are defined as "OA [open access] journals that exist for the sole purpose of profit, not the dissemination of high-quality research findings and furtherance of knowledge." When answering the following questions, use this definition of a predatory journal and only consider open access journals (journals that are freely available with no subscriptions).

2. For each of the following characteristics, indicate whether you would associate it with a legitimate OA journal, predatory journal, neither, or unsure. You can check multiple boxes for each characteristic.

	Legitimate open access journal	Predatory open access journal	Neither	Unsure
Free to read online				
Journal website looks professional				
Rapid acceptance of articles				
Rapid publication of articles				
Presence of a physical mailing address				
Has an article processing fee (authors are required to pay a fee to have their article published)				
Article processing fee seems high				
Article processing fee seems low				

Journal of the Medical Library Association

Supplemental content to
J Med Libr Assoc. Apr;108(2):dx.doi.org/10.5195/jmla.2020.849
www.jmla.mlanet.org
© Swanberg, Thielen, Bulgarelli 2020

	Legitimate open access journal	Predatory open access journal	Neither	Unsure
Has an International Standard Serial Number (ISSN)				
Has an editor and editorial board				
Affiliation and contact information for editor and/or editorial board members provided				
Requires transfer of author copyright prior to publication				
Journal is included in major databases (i.e., PubMed, Scopus, JSTOR, PsycINFO, etc.)				
Listed in the Directory of Open Access Journals (DOAJ)				
Journal has a low impact factor				

3. Over the *past month*, how often have you received solicitations (via email, mail, or phone) to submit an article to a predatory journal?

- Once a day (1)
- A few times per week
- A few times per month
- Never
- Unsure

4. Select the field of study in which you would feel the most comfortable in assessing the quality of a journal:

- Arts
- Humanities
- Medicine and health sciences
- Sciences
- Social sciences

JMLA Journal of the Medical Library Association

SC
Supplemental content to
J Med Libr Assoc. Apr;108(2):dx.doi.org/10.5195/jmla.2020.849
www.jmla.mlanet.org
© Swanberg, Thielen, Bulgarelli 2020

[Survey branches into four sections based on response to question #4]

5. The screenshot below shows the home page of the [insert journal name for humanities and social sciences branch] website. Take a few minutes to review and evaluate the journal website by clicking the following link: [insert journal website uniform resource locator (URL)]. You will then be asked to decide if this journal is predatory.

5. The screenshot below shows the home page of the [insert journal name for medicine and health sciences branch] website. Take a few minutes to review and evaluate the journal website by clicking the following link: [insert journal website URL]. You will then be asked to decide if this journal is predatory.

5. The screenshot below shows the home page of the [insert journal name for sciences branch] journal website. Take a few minutes to review and evaluate the journal website by clicking the following link: [insert journal website URL]. You will then be asked to decide if this journal is predatory.

5. The screenshot below shows the home page of the [insert journal name for arts branch] website. Take a few minutes to review and evaluate the journal website by clicking the following link: [insert journal website URL]. You will then be asked to decide if this journal is predatory.

6. In your opinion, is this open access journal legitimate or predatory?

- Legitimate
- Predatory
- Unsure

7. Provide *at least two reasons* why you believe the journal is legitimate:

- Reason 1: ________________________________
- Reason 2: ________________________________
- Reason 3: ________________________________
- Reason 4: ________________________________

7. Provide *at least two reasons* why you believe the journal is predatory

- Reason 1: ________________________________
- Reason 2: ________________________________
- Reason 3: ________________________________
- Reason 4: ________________________________

7. Provide *at least two reasons* why you are unsure if the journal is legitimate or predatory

- Reason 1: ________________________________
- Reason 2: ________________________________
- Reason 3: ________________________________
- Reason 4: ________________________________

8. Which of the following roles has a predatory journal approached you about? (check all that apply)

- Publish in their journal
- Serve on the editorial board
- Act as a peer reviewer
- None
- Unsure

SC
Supplemental content to
J Med Libr Assoc. Apr;108(2):dx.doi.org/10.5195/jmla.2020.849
www.jmla.mlanet.org
© Swanberg, Thielen, Bulgarelli 2020

9a. Have you ever published in a predatory journal?

- Yes
- No
- Unsure

9b. If you believe you have published in a predatory journal, why did you publish in it? (check all that apply)

- Unaware of it being a predatory journal
- Pressure to publish
- Not confident in ability to publish in high-quality journals
- Could not get published elsewhere
- Option for rapid acceptance and/or publication
- Other (please specify)

10. Please rate your agreement with the following statements

	**Strongly agree**	**Agree**	**Disagree**	**Strongly disagree**	**Unsure**
I feel confident in my ability to assess journal quality					
I know who to ask when I have questions about assessing journal quality					
I feel it is valuable to discuss how to assess journal quality with my colleagues and students					
I believe promotion and tenure review committees should be concerned about predatory journals					
I believe my field should be concerned about predatory journals					

11. Why do you feel confident in your ability to assess journal quality? [freet text]

11. Why do you not feel confident in your ability to assess journal quality? [free text]

12. Have you previously received any training on predatory journals?

- Yes
- No
- Unsure

SC
Supplemental content to
J Med Libr Assoc. Apr;108(2):dx.doi.org/10.5195/jmla.2020.849
www.jmla.mlanet.org
© Swanberg, Thielen, Bulgarelli 2020

13. What type of training did you receive? (check all that apply)

- Library workshop, training, or presentation at Oakland University (OU) or Oakland University William Beaumont School of Medicine (OUWB) Library
- Library workshop, training, or presentation at another institution
- Department/unit workshop, training, or presentation
- Professional conference
- Coursework
- Webinar
- Other (please specify)

14. Currently, what resources do you use to assess journal quality? (check all that apply)

- Colleagues
- Mentors
- Dean of my school/college
- Chair of my department/unit
- Librarian
- Google or other search engine
- Professional email discussion list/blog/website
- OU Libraries' website
- Other (please specify)

15. How can OU Libraries assist you in this area? (check all that apply)

- Individual or small group consultations
- Workshops, trainings, or presentations
- Information on OU Libraries' website
- Checklist to determine whether a journal is legitimate or predatory
- OU Libraries are not helpful in this area
- Other (please specify)

16. Are there other comments you would like to share? [Free text]

17. What is your OU faculty affiliation?

- Oakland University
- Oakland University William Beaumont School of Medicine

18. What is your general area of study/research?

- Arts
- Business
- Engineering and computer science
- Health sciences
- Humanities
- Library science
- Medicine
- Nursing
- Sciences
- Social and behavioral science
- Other (please specify)

JMLA Journal of the Medical Library Association

SC
Supplemental content to
J Med Libr Assoc. Apr;108(2):dx.doi.org/10.5195/jmla.2020.849
www.jmla.mlanet.org
© Swanberg, Thielen, Bulgarelli 2020

18. What is your primary OUWB department affiliation?

- Anesthesiology
- Foundational medical studies (OU-based)
- Foundational medical studies (Beaumont-based)
- Emergency medicine
- Family medicine and community health
- Internal medicine
- Neurology
- Neurosurgery
- Obstetrics and gynecology
- Ophthalmology
- Orthopaedic surgery
- Pathology
- Pediatrics
- Physical medicine and rehabilitation
- Psychiatry
- Radiation oncology
- Diagnostic radiology and molecular imaging
- Surgery
- Urology

19. What is your current academic rank?

- Distinguished professor
- Professor
- Associate professor
- Assistant professor
- Adjunct professor
- Adjunct associate professor
- Adjunct assistant professor
- Adjunct instructor
- Instructor
- Visiting professor
- Researcher
- Emeritus professor
- Other (please specify)

20. During your entire academic career, how many peer-reviewed journal articles have you been an author on?

- 0–5
- 6–10
- 11–20
- 21–30
- 31–50
- 51–99
- 100+
