# Supplementary material for: Faculty knowledge and attitudes regarding predatory open access journals: a needs assessment study
Source: J Med Libr Assoc. 2020 Apr 1;108(2):208–18. doi: 10.5195/jmla.2020.849 (PMC7069810; doi:10.5195/jmla.2020.849)
Supplement: Appendix B [file jmla-108-208-s002.pdf]

JMLA Journal of the Medical Library Association

SC
Supplemental content to
J Med Libr Assoc. Apr;108(2):dx.doi.org/10.5195/jmla.2020.849
www.jmla.mlanet.org
© Swanberg, Thielen, Bulgarelli 2020

Faculty knowledge and attitudes regarding predatory open access journals: a needs assessment study

Stephanie M. Swanberg, MSI, AHIP; Joanna Thielen, MSI, MS; Nancy Bulgarelli, MSLS

APPENDIX B

Supplemental tables

Table S1 Faculty rank reported by respondents who elected to answer question (n=160)

	University faculty		Medical school faculty	
	n	%	n	%
Distinguished professor	4	3.9%	0	–
Professor	12	11.7%	15	26.3%
Associate professor	43	41.7%	15	26.3%
Assistant professor	26	25.2%	17	29.8%
Adjunct professor	1	1.0%	2	3.5%
Adjunct assistant professor	0	–	3	5.3%
Adjunct instructor	4	3.9%	0	–
Instructor	4	3.9%	4	7.0%
Emeritus professor	5	4.9%	0	0.0%
Other	4	3.9%	1	1.8%
Total	103	100%	57	100.0%

JMLA Journal of the Medical Library Association

Supplemental content to
J Med Libr Assoc. Apr;108(2):dx.doi.org/10.5195/jmla.2020.849
www.jmla.mlanet.org
© Swanberg, Thielen, Bulgarelli 2020

Table S2 University faculty respondents by department who elected to answer question (n=103)

	University faculty	
	**n**	**%**
Arts	3	2.9%
Business	10	9.7%
Engineering and computer science	5	4.9%
Health sciences	8	7.8%
Humanities	15	14.6%
Library science	1	0.9%
Nursing	5	4.9%
Sciences	16	15.5%
Social and behavioral science	33	32.0%
Other	7	6.8%
Total	103	100.0%

JMLA Journal of the Medical Library Association

SC

Supplemental content to
J Med Libr Assoc. Apr;108(2):dx.doi.org/10.5195/jmla.2020.849
www.jmla.mlanet.org
© Swanberg, Thielen, Bulgarelli 2020

Table S3 Medical school faculty respondents by department who elected to answer question (n=61)

	**Medical school faculty**	
	**n**	**%**
Diagnostic radiology and molecular imaging	3	4.9%
Emergency medicine	5	8.2%
Family medicine and community health	4	6.6%
Foundational medical studies (university-based)	15	24.6%
Foundational medical studies (hospital based)	4	6.6%
Internal medicine	8	13.1%
Neurology	1	1.6%
Obstetrics and gynecology	1	1.6%
Orthopaedic surgery	3	4.9%
Pathology	2	3.3%
Pediatrics	5	8.2%
Radiation oncology	4	6.6%
Surgery	4	6.6%
Urology	2	3.3%
Total	61	100.0%
